# Expression of p53 Target Genes in the Early Phase of Long-Term Potentiation in the Rat Hippocampal CA1 Area

**DOI:** 10.1155/2015/242158

**Published:** 2015-02-12

**Authors:** Vladimir O. Pustylnyak, Pavel D. Lisachev, Mark B. Shtark

**Affiliations:** ^1^Novosibirsk State University, Pirogova Street 2, Novosibirsk 630090, Russia; ^2^Institute of Molecular Biology and Biophysics SB RAMS, Timakova Street 2, Novosibirsk 630117, Russia; ^3^International Tomography Center (ITC) SB RAS, Institutskaya Street 3-A, Novosibirsk 630090, Russia; ^4^Laboratory of Biomedical Informatics, Design Technological Institute of Digital Techniques SB RAS, Akademika Rzhanova Street 6, Novosibirsk 630090, Russia

## Abstract

Gene expression plays an important role in the mechanisms of long-term potentiation (LTP), which is a widely accepted experimental model of synaptic plasticity. We have studied the expression of at least 50 genes that are transcriptionally regulated by p53, as well as other genes that are related to p53-dependent processes, in the early phase of LTP. Within 30 min after Schaffer collaterals (SC) tetanization, increases in the mRNA and protein levels of Bax, which are upregulated by p53, and a decrease in the mRNA and protein levels of Bcl2, which are downregulated by p53, were observed. The inhibition of Mdm2 by nutlin-3 increased the basal p53 protein level and rescued its tetanization-induced depletion, which suggested the involvement of Mdm2 in the control over p53 during LTP. Furthermore, nutlin-3 caused an increase in the basal expression of Bax and a decrease in the basal expression of Bcl2, whereas tetanization-induced changes in their expression were occluded. These results support the hypothesis that p53 may be involved in transcriptional regulation during the early phase of LTP. We hope that the presented data may aid in the understanding of the contribution of p53 and related genes in the processes that are associated with synaptic plasticity.

## 1. Introduction

The storage of information in the brain relies on long-term synaptic plasticity, which depends on complex molecular interactions involving gene expression. One of the forms of synaptic plasticity is hippocampal long-term potentiation (LTP). The late phase of LTP is known to be dependent on mRNA and protein synthesis during a brief time after stimulus [1].

Several transcription factors are rapidly induced in association with LTP [2]. We demonstrated earlier that the tetanization of SC in rat hippocampal slices, which induces the long-term potentiation of CA3-CA1 synapses, is accompanied by a brief (less than 40 min) increase in the binding of transcription factor p53 with the promoter of S100B and by an increase in the level of S100B mRNA [3]. Interestingly, the maximal increase in the DNA-binding activity of p53 coincided with the maximal rate of decrease in the p53 protein level, which suggested the activation of negative feedback to p53.

p53 is a key regulator of the cell cycle and “programmed cell death” (apoptosis). Biological functions of p53 are primarily mediated through the transcriptional regulation of target genes [4, 5]. Under stress conditions, the increased activity of р53 can increase the susceptibility of cells to death signals by shifting the balance between proapoptotic (Bax, Noxa, and Puma) and antiapoptotic (Bcl2, Birc5) proteins of the Bcl2 family, which regulate the activity of proteolytic enzymes called caspases. In addition, p53 can induce the expression of “death receptors,” which initiate apoptosis by the binding of their cognate ligands. Thus, upon activation, р53 can induce apoptosis by the activation of caspases through multiple mechanisms [6].

p53 is widely known primarily due to its ability to suppress tumors; however, the list of its functions is growing. Accumulating evidence suggests that p53 should be viewed as a crucial decision-maker molecule rather than as a tumor suppressor protein [5, 7]. p53, caspases, and Bcl-2 family members can regulate the proliferation and differentiation of neural progenitor cells, as well as neurite outgrowth and regeneration [7, 8]. The activation of caspases, which is regulated by Bcl2 family proteins, seems to be necessary for synaptic modifications during long-term depression [9] and to contribute to LTP [10]. Another p53 transcriptional target, microRNA-34a, also regulates neurite outgrowth, spinal morphology, and function [11]. Finally, p53 regulates the transcription of genes that encode secreted proteins, such as interleukin 6, TNF-*α*, and S100B, which can modulate synaptic plasticity [12, 13].

Thus, which functional consequences might the brief activation of p53 have? The control of the transcriptional activity of p53 is considered crucial for determining which p53 response is activated [5]. In this paper, we describe the expression of p53-related genes in the rat hippocampal CA1 area in the early phase of long-term potentiation (30 min after tetanization) using real-time PCR. Our set of genes includes at least 50 genes that are transcriptionally regulated (directly or indirectly) by p53, as well as other p53-related genes, which are not known p53 transcriptional targets. The posttetanization time point was chosen from our previous data on the tetanization-induced increase in S100B mRNA, which is maximal at 30 min after tetanization [3, 14].

p53 transcriptional targets are regulated by multiple factors. One of the tools for the preliminary assessment of the transcription dependency on p53 is the inhibition of Mdm2 by nutlin-3 [15–17]. Mdm2 negatively modulates the transcriptional activity, the stability, and the mRNA translation of p53, and the inhibition of Mdm2 results in the activation of p53 and the increase in the p53 protein level [13, 18–20]. Therefore, to estimate the contribution of p53 to the tetanization-induced regulation of the genes studied, we reexecuted our experiments using nutlin-3.

Although not definitive, our results allow hypothesizing that p53 participates in transcriptional regulation during the early phase of LTP. We hope that the presented data may aid in the understanding of the physiological function of p53-related genes in the processes that are associated with synaptic plasticity.

## 2. Materials and Methods

### 2.1. Experimental Animals

Male Wistar rats (7–9 weeks of age) were supplied by the Institute of Cytology and Genetics SB RAS (Novosibirsk, Russia). Animals were acclimated for 1 week and were allowed free access to food and water. All experimental procedures were approved by the Animal Care Committee for the Institute of Molecular Biology and Biophysics SB RAMS and were performed in strict accordance with the National Institutes of Health guidelines.

### 2.2. Hippocampal Slice Preparation and Tetanization

Animals were decapitated, and the brain was rapidly removed and placed in ice-cold oxygenated (95% O_2_, 5% CO_2_) artificial cerebrospinal fluid (ACSF): 126 mM NaCl, 4 mM KCl, 1.24 mM NaH_2_PO_4_, 1.3 mM MgSO_4_, 2 mM CaCl_2_, 26 mM NaHCO_3_, and 10 mM D-glucose, pH 7.4. The left hippocampus was dissected for removal and cut into 400 *μ*m thick transverse slices, using a chopper. Four consecutive slices from the dorsal region of the hippocampus were transferred to a submerged recording chamber. Slices were perfused at a rate of 2 mL/min with fresh, oxygenated ACSF at room temperature (22–24°C) for 30 min; then the perfusion rate was reduced to 1.5 mL/min, and the temperature was raised to 32-33°C. For extracellular recordings, the recording electrode, which was filled with ACSF, was placed in the CA1 pyramidal cell layer. To stimulate Schaffer collaterals, stimulating electrodes, which were filled with ACSF, were placed in the stratum radiatum. The intensity of the stimulation was adjusted to obtain a p-spike amplitude that was ~50% of the maximal response.

P-spikes amplitudes were measured as the vertical distance from the peak of the spike to the line joining the peak positivities on either side. Slopes of field excitatory postsynaptic potentials were measured as the slope of the line joining the peak of the presynaptic volley and the peak positivity of the response.

Racemic nutlin-3 (Sigma-Aldrich) was dissolved in DMSO and stored at –20°C. Stock aliquots were dissolved in ACSF ex temporo. Slices were perfused with ACSF, which contained nutlin-3 (20 *μ*M) or vehicle (DMSO 0.1%), from 30 min before to 30 min after the onset of tetanization.

In the experiments that were intended for the preparation of samples for real-time PCR and Western blot analysis, two of the four slices remained nonstimulated throughout the entire incubation period (4 h 15 min) and were used as a baseline control in the corresponding set of samples. Two other slices were tetanized (4 trains of 1 s, 100 Hz stimulations spaced by 30 s intervals) 30 min before the termination of incubation. Electrodes were placed on the slices 2–5 min before the tetanization and were removed immediately after the stimulation. At the end of the incubation, slices were transferred to ice-cold oxygenated ACSF, and the CA1 field was rapidly cut away from each slice, as described previously [14], and placed into the Allprotect Tissue Reagent (Qiagen). Six relevant slices from three animals were pooled to prepare one mRNA/protein sample. Four independent tetanization/control pairs of samples were prepared for each (nutlin-3/vehicle) incubation condition.

### 2.3. cDNA Synthesis and Real-Time PCR

To identify the participation of p53 in transcriptional regulation during the early phase of LTP, a Rat p53 Signaling Pathway RT² Profiler PCR Array (Qiagen) was used. Total RNA was isolated using an AllPrep DNA/RNA/Protein Mini Kit (Qiagen) according to manufacturer's protocol. The RNA concentration was measured using a Quant-iT Assay Kit and a Qubit instrument (Invitrogen); the RNA quality was evaluated by electrophoresis. cDNA was synthesized from total RNA using RT² First Strand cDNA Synthesis Kit (Qiagen). Real-time PCR was performed with a Maxima SYBR Green qPCR Master Mix (Thermo Scientific) and a CFX96 Multicolor Detection System (Bio-Rad) according to manufacturer's instructions. The obtained data were analyzed using the Excel-based PCR Array Data Analysis Software (Qiagen). The mean of Ct values of five housekeeping genes (Actb, *β*-actin; B2m, beta-2 microglobulin; Hprt1, hypoxanthine phosphoribosyltransferase 1; Ldha, lactate dehydrogenase A; Rplp1, ribosomal protein, large, P1) was used as the reference according to manufacturer's instructions. The concentration of S100B mRNA was measured as described previously [68].

### 2.4. Preparation of Whole-Cell Extracts

Whole-cell extracts from frozen slices were prepared using an AllPrep DNA/RNA/Protein Mini Kit (Qiagen) according to the manufacturer's protocol. Protein concentrations were determined using a Quant-iT Assay Kit and a Qubit instrument (Invitrogen).

### 2.5. SDS-PAGE Electrophoresis and Western Blot

Sixty micrograms of whole-cell proteins per lane was separated using 12% SDS-PAGE and transferred to nitrocellulose membrane. Membranes were stained with Ponceau S to verify the loading and transfer efficiency. Immunodetection was performed using anti-p53 (1 : 500, Santa Cruz Biotechnology), anti-bax (1 : 2000), and anti-bcl2 (1 : 1000, Abcam) or anti-*β*-actin (1 : 2000, Sigma-Aldrich) antibodies. *β*-actin was used as a control to ensure the equal loading of samples. The bands were visualized using Visualizer Spray and a Glow ECL Western Blotting Detection System (Millipore).

### 2.6. Data Analysis

The data are expressed as the mean ± S.E.M. The significance was assessed using Student's *t*-tests with the criterion set at *P* < 0.05. In order to exclude the overestimation of the significance of studied genes mRNA fold changes due to possible inappropriate biases in values of housekeeping genes mRNAs, we performed the additional statistical analysis based on calculations made on the assumption that the expression of the housekeeping genes is constant. mRNA fold changes were considered as significant, if they were significant in both standard and additional tests. Besides, fold changes, which ranged from 0.95 to 1.05, were considered as not significant.

## 3. Results

**Figure 1:**
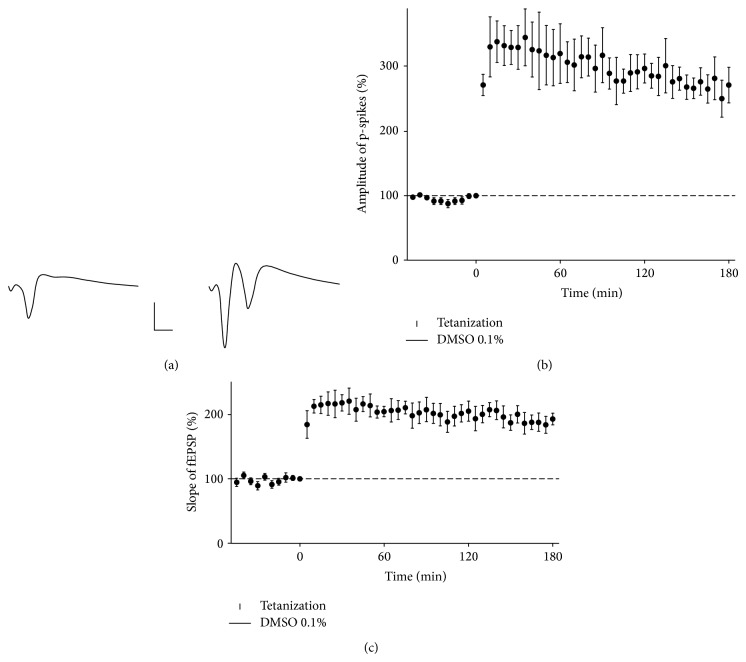
Long-term potentiation in the rat hippocampal CA1 area. (a) Representative responses to test stimuli before tetanization (left) and 30 min after tetanization (right). Calibration bars: 3 ms, 1 mV. (b, c) Abscissa, the time from the onset of tetanization. Ordinate, the amplitude of p-spikes (b) or the slope of field excitatory postsynaptic potentials (c), which is normalized to the amplitude of the response to the stimulus that immediately preceded tetanization. The data are the mean ± S.E.M. (*n* = 4).

We used real-time PCR analysis to study the expression of 85 genes that are functionally related to p53 in the early phase of LTP in the CA1 area of rat hippocampal slices (Table 1). As described earlier [3, 69], our experimental protocol induces robust and enduring potentiation slightly reducing at 3 h after tetanization, which is characteristic of late-LTP produced in rat CA1 by repeated tetanization [70]. The application of vehicle (DMSO 0.1%) in perfusing milieu from 30 min before to 30 min after tetanization did not influence significantly the basal responses (Figure 1), and the potentiation time course also did not differ significantly from that which has been previously described. Preliminary experiments showed that DMSO had no effect on basal expressions and tetanization-induced mRNA fold changes of genes studied (not presented).

A short description of the genes studied is presented in Table 2. A variant of their functional classification is available on the PCR array manufacturer's website (http://www.sabiosciences.com/rt_pcr_product/HTML/ PARN-027Z.html). Four transcripts (LOC367198, Myod1, Serpinb5, and Wt1) were not detected in our samples (Ct > 35) and, therefore, were omitted in Table 1. Besides, Esr1, Lig4, Sfn, and Tnfrsf10b are not presented, since their changes were not significant in all the comparisons.

The tetanization-induced changes in expression profiles of genes that are upregulated (p53-URG) or downregulated by p53 (p53-DRG) did not differ significantly. Specifically, the expression of only two p53-DRGs (11%), Bcl2 and Pttg1, significantly decreased, whereas the expression of seven p53-DRGs (37%) significantly increased after tetanization. Similarly, the expression of two p53-URGs (6%), Bbc3/Puma and Rprm, also decreased, whereas the expression of 18 p53-URGs (58%) increased after tetanization. Thus, the LTP-related regulation of p53 transcriptional target genes appears to be rather complex, and the contribution of p53 to this regulation seems to not be crucial, in many instances.

To estimate the possible contribution of p53 to the regulation of tetanization-induced gene expression, we inhibited Mdm2 by nutlin-3. Surprisingly, nutlin-3 attenuated basal mRNA levels of only a few p53-DRGs (Bcl2, Prkca, and Pttg1), whereas the expression of most genes increased (Table 1), which partly might be associated with the involvement of Mdm2 in the regulation of mRNA stability [71].

**Figure 2:**
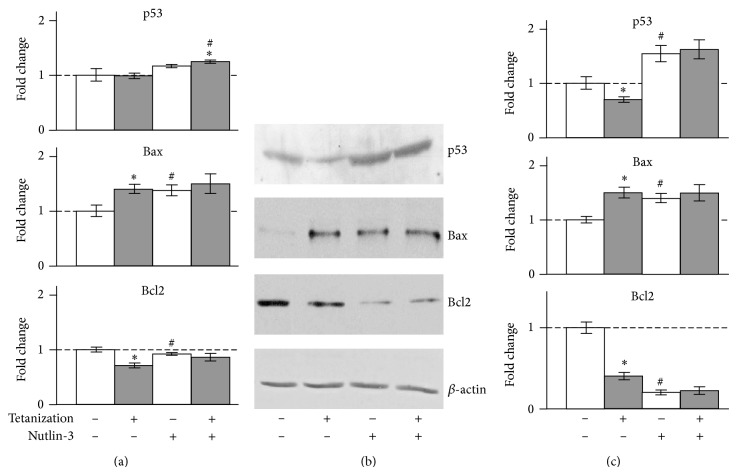
Effect of tetanization on p53, Bax, and Bcl2 in the rat hippocampal CA1 area. (a) Total RNAs were prepared and subjected to real-time PCR for the measurement of mRNAs. The mean of Ct values of five housekeeping genes was used as internal control for normalization as described in Section 2. (b) Representative Western blots. Whole-cell extracts were prepared from the rat hippocampal CA1 area and subjected to Western blot analysis as described in Section 2. (c) Relative intensity. The protein bands were analyzed by the computerized densitometric program “Total Lab.” The intensities of the signals were determined from the areas under the curves for each peak and data were graphed. *β*-actin was used as internal control for normalization. The fold changes were expressed by taking the average value of the group tetanization^(−)^/nutlin-3^(−)^ as one. ^*^ *P* < 0.05 against corresponding tetanization^(−)^ samples (paired *t*-test), ^#^ *P* < 0.05 against the group tetanization^(−)^/nutlin-3^(−)^ (*t*-test), *n* = 4.

We assumed that necessary (but not sufficient) criteria for a gene, which is regulated predominantly by p53 after tetanization, are (1) the level of mRNA changes significantly after tetanization under normal conditions; (2) nutlin significantly shifts the basal expression in the same direction as tetanization; and (3) in the presence of nutlin, the effect of tetanization is not significant. We found 16 genes, which conformed to these criteria (Table 1). Six of them (Bax, Bcl2, Cdkn1a, Gadd45a, Mdm2, and Pten) are known p53 target genes and, therefore, might be considered as promising candidates for more detailed studies of the possible involvement of p53 in the tetanization-induced transcriptional regulation. We chose two of these genes, proapoptotic Bax and antiapoptotic Bcl2, that are up- and downregulated by p53, respectively, to confirm the changes in their expression on the protein level. Indeed, Bax and Bcl2 protein contents followed changes in the mRNA contents (Figure 2).

Consistent with previous results [3], the induction of LTP was accompanied by a decrease in the protein level of p53 (Figures 2(b) and 2(c)). Nutlin-3 increased the average basal p53 protein level and completely blocked its tetanization-induced depletion (Figures 2(b) and 2(c)). This effect seemed to be associated not only with the inhibition of the Mdm2-dependent degradation of p53 but also with its augmented synthesis due to elevated levels of p53 mRNA (Figure 2(a), Table 1).

## 4. Discussion

In spite of the remarkable achievements of many researchers in the identification of LTP-related genes, the extremely complex transcriptional program that is activated in neuroglial networks during LTP remains poorly understood. Our previous results [3] suggest that LTP in the hippocampal CA1 area is accompanied by a transient increase in the transcriptional activity of p53. However, we could find only a few reported examples of p53 target genes that are transcriptionally regulated during the early phase of hippocampal LTP and only in the dentate gyrus [72–75]. Hippocampal subregions differ one from another in their gene expression profiles [76]. Besides, gene expression differences across the septotemporal (dorsal-ventral) axis of the hippocampus within CA1 were demonstrated [77], and the induction of LTP is differently regulated in dorsal hippocampus versus ventral hippocampus [78]. Therefore, we studied the expression of p53-related genes in the early phase of LTP in the rat dorsal hippocampal CA1 area using real-time PCR analysis. Our set of genes was composed of at least 50 genes that are transcriptionally regulated by p53 (directly or indirectly), as well as other genes that are related to p53-dependent processes (Tables 1 and 2).

To evaluate the contribution of p53 to tetanization-induced expression profile changes, we inhibited the primary negative regulator of p53 Mdm2 by nutlin-3. Mdm2 negatively modulates (partly independently) the transcriptional activity, the stability, and the mRNA translation of p53, and the inhibition of Mdm2 by nutlin results in both the increase in p53 protein level and the activation of p53-dependent transcription [15]. Therefore, we measured the protein level of p53 to assess the effectiveness of nutlin-3 under our experimental conditions. We observed the significant increase in the protein level of p53 in the presence of nutlin-3, which suggested the effective inhibition of Mdm2 and, therefore, the transcriptional activation of p53. However, because Mdm2 may also influence the transcription of p53 targets through p53-independent pathways [20, 71], the results that were obtained using nutlin-3 are only circumstantial and are not definitive.

When LTP is induced, the activity of p53 cannot correlate with its total protein amount [3], which is not amazing. The activity of p53 is regulated by numerous mechanisms [5, 79], and the induction of LTP is associated with the activation of multiple regulatory cascades [1, 2, 80], which might influence the portion of the activated p53, its localization, and the recruitment of cofactors.

We observed the upregulation of numerous p53 targets, as well as other p53-related genes, at 30 min after tetanization. However, a percentage of the upregulated genes in the p53-URG group insignificantly exceeded that in the p53-DRG group. Therefore, it is evident, for p53-DRGs and, by analogy, extremely likely for p53-URGs, that p53 is not a single mediator of their LTP-associated regulation. The p53 homologue p73 represents an example of such a complex regulation. Tp73 is one of the genes that are upregulated the most after tetanization in our samples. However, this gene can be upregulated not only by p53 but also by p73 itself and by Egr1 [40]. Nutlin-3 only partly simulated the tetanization-induced upregulation of Tp73 and did not effectively occlude the effect of tetanization (Table 1), which suggests the relatively small involvement of p53 in tetanization-induced upregulation of Tp73.

Egr1 is an immediate early gene, which appears to be critical for memory formation and LTP maintenance [2]. However, in response to stress, Egr1 displays a remarkable functional similarity to p53 and p73 [81–84]. Egr1 target genes overlap those genes of p53. Egr1, p73, and p53 form a network with positive feedback loops, which respond to stress by the prolonged expression of the p53 family of genes, which results in efficient apoptosis [40]. Our results indicate that the activation of this network is strongly restricted during LTP by Mdm2 activity. Specifically, the induction of LTP was followed by a decrease in the p53 protein level, whereas the p53 mRNA level did not change. However, when Mdm2 was inhibited by nutlin-3 not only was the p53 protein level rescued but also p53 mRNA increased at 30 min after tetanization. Thus, Mdm2 seems to effectively attenuate the activity of the Egr1-p73-p53 network during LTP, which might be explained by the capability of Mdm2 to suppress the transcriptional function of both p53 and p73 [85].

## 5. Conclusion

Thus, during the formation of LTP in the hippocampal CA1 area, the increase in the transcriptional activity of p53 seems to occur under tight constraints and leads to the selective transcriptional regulation of target genes. The induction of LTP entails the transcriptional upregulation and/or posttranslational activation of negative regulators of p53 (such as Mdm2), which lead to a rapid cessation of p53 activity. Moreover, in some instances, p53 activity is overridden by other neuron activity-dependent transcriptional regulators. The definite functional roles of p53 and related factors in tetanization-induced processes remain to be elucidated, and now the relevance of these factors to excitotoxicity or to abnormal in vitro conditions cannot be excluded. Nevertheless, it is possible that p53-dependent transcriptional program may be an essential part of synaptic activity-driven adaptive processes. Our results may help to understand the physiological function of p53 pathway in the processes associated with synaptic plasticity. However, to further corroborate the functional significance of p53 in transcriptional regulation during LTP, more detailed studies should be performed using chromatin immunoprecipitation, p53-knockdown or mRNA interference models, promoter constructs, and immunohistochemistry.

## Figures and Tables

**Table 1:** Expression of p53-related genes in rat hippocampal CA1 area 30 min after tetanization.

Symbol	p53 transcriptional target genes		Vehicle		Nutlin-3			
			Tetanization-induced fold change		Basal expression fold change		Tetanization-induced fold change	
	Regulation	Reference	TSv/CSv	Paired *t*-test, *P* < 0.05	CSn/CSv	*t*-test, *P* < 0.05	TSn/CSn	Paired *t*-test, *P* < 0.05
Apaf1	+	[21, 22]	1,25	∗	1,25	∗	1,25	∗
*Apex1 *			1,11	∗	1,29	∗	0,98	ns
Atm			1,27	∗	1,37	∗	1,08	∗
*Bag1 *			1,13	∗	1,30	∗	1,01	ns
*Bax *	+	[23]	1,41	∗	1,38	∗	1,08	ns
Bbc3	+	[24]	0,93	∗	1,33	∗	0,87	∗
*Bcl2 *	−	[23]	0,71	∗	0,92	∗	0,94	ns
Bid	+	[25]	1,26	∗	1,23	∗	1,19	∗
Birc5	−	[26]	1,22	ns	1,35	∗	1,08	ns
Bnip3			1,13	∗	1,27	∗	1,08	∗
Brca1	−	[27]	1,38	∗	1,58	∗	1,51	ns
Brca2			1,20	∗	1,10	∗	1,51	∗
Btg2	+	[28]	1,47	∗	1,41	∗	1,11	∗
Casp2			1,30	∗	1,34	∗	1,12	∗
*Casp9 *			1,14	∗	1,28	∗	1,02	ns
Ccnb1	−	[29]	1,03	ns	1,12	∗	1,18	∗
*Ccne1 *			1,26	∗	1,31	∗	1,10	ns
Ccng1	+	[30]	1,31	∗	1,35	∗	1,16	∗
Ccnh			1,18	∗	1,25	∗	1,11	∗
Cdc25a	−	[31]	1,09	ns	1,06	ns	1,31	∗
Cdc25c	−	[32]	1,17	ns	1,13	∗	1,27	∗
Cdk1	−	[33]	0,96	ns	1,01	ns	1,31	∗
Cdk4			1,10	∗	1,24	∗	1,07	∗
*Cdkn1a *	+	[34]	1,20	∗	1,17	∗	1,15	ns
Cdkn2a	−	[35]	1,06	ns	1,38	∗	1,19	ns
*Chek1 *	−	[27]	1,31	∗	1,29	∗	1,07	ns
Chek2	−	[36]	1,04	ns	1,22	ns	1,18	∗
Cul9			1,20	ns	1,35	∗	1,05	ns
Dapk1	+	[37]	1,34	∗	1,41	∗	0,90	∗
Dnmt1	**−**	[38]	1,15	ns	1,19	ns	1,16	∗
E2f1	−	[17]	1,34	∗	1,22	∗	1,28	∗
E2f3			1,62	∗	1,78	∗	0,76	∗
Egfr	+	[39]	1,39	∗	1,33	∗	1,29	∗
Egr1	+	[40]	1,22	∗	1,21	∗	1,11	∗
*Ep300 *			1,17	∗	1,22	∗	1,02	ns
Ercc1			1,17	∗	1,29	∗	1,11	∗
Fadd			1,39	∗	1,57	∗	0,83	∗
Fas	+	[41, 42]	1,18	ns	1,14	∗	1,23	ns
Faslg	+	[43]	1,19	ns	1,19	∗	1,14	∗
Foxo3			1,49	∗	1,69	∗	0,79	∗
*Gadd45a *	+	[44]	1,26	∗	1,41	∗	0,96	ns
Hdac1			1,12	ns	1,24	∗	1,10	ns
*Hif1a *			1,30	∗	1,46	∗	0,95	ns
Igf1r			1,08	ns	1,14	∗	1,11	∗
Il6	−	[45]	1,45	∗	1,31	∗	1,26	∗
Jun			1,35	∗	1,32	∗	1,15	∗
Kras			1,11	ns	1,22	∗	1,07	ns
Mcl1			1,30	∗	1,32	∗	1,12	∗
*Mdm2 *	+	[46]	1,21	∗	1,22	∗	1,13	ns
Mdm4			1,16	ns	1,27	∗	1,03	ns
Mlh1	**+**	[47]	1,16	ns	1,21	∗	1,06	ns
Msh2	+	[48]	1,10	ns	1,11	ns	1,16	∗
Myc	−	[49]	1,48	∗	1,31	∗	1,34	∗
Nf1			1,54	∗	1,25	∗	1,37	∗
Nfkb1			1,27	∗	1,27	∗	1,12	∗
Pcna	**+**	[50]	1,33	∗	1,28	∗	1,29	∗
Pmaip1	+	[51]	1,55	∗	1,47	∗	1,16	∗
Ppm1d	+	[52]	1,11	∗	1,23	∗	1,10	∗
Prc1	−	[53]	1,29	ns	1,28	∗	1,20	ns
Prkca	−	[54]	1,30	∗	0,92	∗	1,58	∗
*Pten *	+	[55]	1,47	∗	1,46	∗	0,92	ns
Pttg1	−	[56]	0,89	∗	0,96	ns	0,69	∗
Rb1	−	[57]	1,16	ns	1,30	∗	1,01	ns
*Rela *			1,18	∗	1,31	∗	0,99	ns
RGD1566319	+	[58]	1,23	ns	1,32	∗	1,04	ns
Rprm	+	[59]	0,74	∗	0,95	ns	1,15	∗
S100B	+	[60]	2,93	∗	1,51	∗	1,88	∗
Sirt1			1,46	∗	1,32	∗	1,22	∗
*Stat1 *			1,24	∗	1,36	∗	1,00	ns
Tnf	+	[61]	1,28	∗	1,25	∗	1,13	∗
Tp53	+	[40, 62]	1,00	ns	1,17	ns	1,07	∗
*Tp53bp2 *	−	[63]	1,11	∗	1,13	∗	1,06	ns
Tp63	**+**	[64]	1,17	ns	0,98	ns	1,39	∗
Tp73	+	[40, 65, 66]	2,15	∗	1,43	∗	1,76	∗
Xrcc4			1,03	ns	1,14	∗	1,15	∗
Xrcc5			1,35	ns	1,40	∗	1,10	ns
Zmat3	+	[67]	0,99	ns	1,06	ns	1,22	∗

TSv, CSv, TSn, CSn: test and control samples from experiments with vehicle (DMSO 0.1%) or nutlin-3 (20 *μ*M). Tetanization-induced fold changes (TSv/CSv and TSn/CSn) were expressed by taking the value of the corresponding control as one. Basal expression fold change (CSn/CSv) was expressed by taking average value of the group CSv as one. Italic marks symbols of the genes, which are regulated similarly by tetanization and nutlin, and effect of tetanization is occluded by nutlin. ^*^ *P* < 0.05 (*n* = 4), ns: not significant.

**Table 2:** Description of p53-related genes.

RefSeq	Symbol	Description	Gene name
NM_023979	Apaf1	Apoptotic peptidase activating factor 1	—
NM_024148	Apex1	APEX nuclease (multifunctional DNA repair enzyme) 1	APE, Apex, REF-1
NM_001106821	Atm	Ataxia telangiectasia mutated homolog (human)	—
NM_001106647	Bag1	BCL2-associated athanogene	—
NM_017059	Bax	Bcl2-associated X protein	—
NM_173837	Bbc3	Bcl-2 binding component 3	Puma
NM_016993	Bcl2	B-cell CLL/lymphoma 2	Bcl-2
NM_022684	Bid	BH3 interacting domain death agonist	—
NM_022274	Birc5	Baculoviral IAP repeat-containing 5	AP14
NM_053420	Bnip3	BCL2/adenovirus E1B interacting protein 3	MGC93043
NM_012514	Brca1	Breast cancer 1	—
NM_031542	Brca2	Breast cancer 2	—
NM_017259	Btg2	BTG family, member 2	Agl, An, Pc3, Tis21, an-1
NM_022522	Casp2	Caspase 2	—
NM_031632	Casp9	Caspase 9, apoptosis-related cysteine peptidase	Apaf3, Casp-9-CTD, Casp9_v1, Ice-Lap6, Mch6
NM_171991	Ccnb1	Cyclin B1	—
NM_001100821	Ccne1	Cyclin E1	CYCLE, Ccne
NM_012923	Ccng1	Cyclin G1	CYCG, Ccng, MGC93642
NM_052981	Ccnh	Cyclin H	—
NM_133571	Cdc25a	Cell division cycle 25 homolog A (*S. pombe*)	—
NM_001107396	Cdc25c	Cell division cycle 25 homolog C (*S. pombe*)	—
NM_019296	Cdk1	Cyclin-dependent kinase 1	Cdc2, Cdc2a
NM_053593	Cdk4	Cyclin-dependent kinase 4	—
NM_080782	Cdkn1a	Cyclin-dependent kinase inhibitor 1A	Cip1, Waf1
NM_031550	Cdkn2a	Cyclin-dependent kinase inhibitor 2A	Arf, INK4A, MTS1, p16, p16Cdkn2a, p19ARF
NM_080400	Chek1	CHK1 checkpoint homolog (*S. pombe*)	—
NM_053677	Chek2	CHK2 checkpoint homolog (*S. pombe*)	Chk2, Rad53
XM_236927	Cul9	Cullin 9	Parc, RGD1562008
NM_001107335	Dapk1	Death associated protein kinase 1	—
NM_053354	Dnmt1	DNA (cytosine-5-)-methyltransferase 1	—
NM_001100778	E2f1	E2F transcription factor 1	—
NM_001137626	E2f3	E2F transcription factor 3	RGD1561600
NM_031507	Egfr	Epidermal growth factor receptor	ERBB1, ErbB-1, Errp
NM_012551	Egr1	Early growth response 1	Krox-24, NGFI-A, Ngf1, Ngfi, zif-268
XM_576312	Ep300	E1A binding protein p300	—
NM_001106228	Ercc1	Excision repair cross-complementing rodent repair deficiency, complementation group 1	—
NM_012689	Esr1	Estrogen receptor 1	ER-alpha, Esr, RNESTROR
NM_152937	Fadd	Fas (TNFRSF6) associated via death domain	Mort1
NM_139194	Fas	Fas (TNF receptor superfamily, member 6)	Tnfrsf6
NM_012908	Faslg	Fas ligand (TNF superfamily, member 6)	Apt1Lg1, CD95-L, Fasl, Tnfsf6
NM_001106395	Foxo3	Forkhead box O3	Fkhrl1, Foxo3a
NM_024127	Gadd45a	Growth arrest and DNA-damage-inducible, alpha	Ddit1, Gadd45
NM_001025409	Hdac1	Histone deacetylase 1	—
NM_024359	Hif1a	Hypoxia-inducible factor 1, alpha subunit (basic helix-loop-helix transcription factor)	MOP1
NM_052807	Igf1r	Insulin-like growth factor 1 receptor	IGFIRC, JTK13
NM_012589	Il6	Interleukin 6	ILg6, Ifnb2
NM_021835	Jun	Jun oncogene	—
NM_031515	Kras	V-Ki-ras2 Kirsten rat sarcoma viral oncogene homolog	Kras2, c-Ki-ras, p21
NM_001106095	Lig4	Ligase IV, DNA, ATP-dependent	—
XM_346005	LOC367198	Similar to Serine/threonine-protein kinase ATR (Ataxia telangiectasia and Rad3-related protein)	—
NM_021846	Mcl1	Myeloid cell leukemia sequence 1	—
NM_001108099	Mdm2	Mdm2 p53 binding protein homolog (mouse)	—
NM_001012026	Mdm4	Mdm4 p53 binding protein homolog (mouse)	—
NM_031053	Mlh1	MutL homolog 1 (*E. coli*)	—
NM_031058	Msh2	MutS homolog 2 (*E. coli*)	—
NM_012603	Myc	Myelocytomatosis oncogene	MGC105490, RNCMYC, c-myc, mMyc
NM_176079	Myod1	Myogenic differentiation 1	MGC156574
NM_012609	Nf1	Neurofibromin 1	—
XM_342346	Nfkb1	Nuclear factor of kappa light polypeptide gene enhancer in B-cells 1	NF-kB
NM_022381	Pcna	Proliferating cell nuclear antigen	PCNAR, Pcna, cyclin
NM_001008385	Pmaip1	Phorbol-12-myristate-13-acetate-induced protein 1	Noxa
NM_001105825	Ppm1d	Protein phosphatase 1D magnesium-dependent, delta isoform	—
NM_001107529	Prc1	Protein regulator of cytokinesis 1	—
NM_001105713	Prkca	Protein kinase C, alpha	Pkca
NM_031606	Pten	Phosphatase and tensin homolog	MMAC1, Mmac, TEP1
NM_022391	Pttg1	Pituitary tumor-transforming 1	Pttg
NM_017045	Rb1	Retinoblastoma 1	—
NM_199267	Rela	V-rel reticuloendotheliosis viral oncogene homolog A (avian)	NFkB
NM_001109358	RGD1566319	Similar to Sestrin 2 (Hi95)	—
NM_001044276	Rprm	Reprimo, TP53 dependent G2 arrest mediator candidate	MGC109515
NM_013191	S100B	S100 calcium binding protein B	—
NM_057108	Serpinb5	Serpin peptidase inhibitor, clade B (ovalbumin), member 5	Maspin, PI-5, Pi5
XM_232745	Sfn	Stratifin	—
NM_001107627	Sirt1	Sirtuin (silent mating type information regulation 2 homolog) 1 (*S. cerevisiae*)	Sir2
NM_032612	Stat1	Signal transducer and activator of transcription 1	—
NM_012675	Tnf	Tumor necrosis factor (TNF superfamily, member 2)	MGC124630, RATTNF, TNF-alpha, Tnfa
NM_001108873	Tnfrsf10b	Tumor necrosis factor receptor superfamily, member 10b	—
NM_030989	Tp53	Tumor protein p53	MGC112612, Trp53, p53
XM_223012	Tp53bp2	Tumor protein p53 binding protein, 2	Trp53bp2
NM_019221	Tp63	Tumor protein p63	Ket, P73l, Tp73l, Trp63
NM_001108696	Tp73	Tumor protein p73	P73, Trp73
NM_031534	Wt1	Wilms tumor 1	—
NM_001006999	Xrcc4	X-ray repair complementing defective repair in Chinese hamster cells 4	MGC95022
NM_177419	Xrcc5	X-ray repair complementing defective repair in Chinese hamster cells 5	Ku80, Kup80
NM_022548	Zmat3	Zinc finger, matrin type 3	PAG608, Wig1

## References

[1] Reymann K. G., Frey J. U. (2007). The late maintenance of hippocampal LTP: requirements, phases, ‘synaptic tagging’, ‘late-associativity’ and implications. *Neuropharmacology*.

[2] Alberini C. M. (2009). Transcription factors in long-term memory and synaptic plasticity. *Physiological Reviews*.

[3] Pustylnyak V. O., Lisachev P. D., Shtark M. B., Epstein O. I. (2011). Regulation of S100B gene in rat hippocampal CA1 area during long term potentiation. *Brain Research*.

[4] Laptenko O., Prives C. (2006). Transcriptional regulation by p53: one protein, many possibilities. *Cell Death and Differentiation*.

[5] Vousden K. H., Prives C. (2009). Blinded by the Light: the growing complexity of p53. *Cell*.

[6] Kuribayashi K., El-Deiry W. S. (2008). Regulation of programmed cell death by the p53 pathway. *Advances in Experimental Medicine and Biology*.

[7] Tedeschi A., Di Giovanni S. (2009). The non-apoptotic role of p53 in neuronal biology: enlightening the dark side of the moon. *The EMBO Reports*.

[8] Solá S., Aranha M. M., Rodrigues C. M. P. (2012). Driving apoptosis-relevant proteins toward neural differentiation. *Molecular Neurobiology*.

[9] Li Z., Jo J., Jia J. M. (2010). Caspase-3 activation via mitochondria is required for long-term depression and AMPA receptor internalization. *Cell*.

[10] Gulyaeva N. V., Kudryashov I. E., Kudryashova I. V. (2003). Caspase activity is essential for long-term potentiation. *Journal of Neuroscience Research*.

[11] Agostini M., Tucci P., Steinert J. R. (2011). microRNA-34a regulates neurite outgrowth, spinal morphology, and function. *Proceedings of the National Academy of Sciences of the United States of America*.

[12] Nishiyama H., Knöpfel T., Endo S., Itohara S. (2002). Glial protein S100B modulates long-term neuronal synaptic plasticity. *Proceedings of the National Academy of Sciences of the United States of America*.

[13] McAfoose J., Baune B. T. (2009). Evidence for a cytokine model of cognitive function. *Neuroscience and Biobehavioral Reviews*.

[14] Lisachev P. D., Shtark M. B., Sokolova O. O., Pustylnyak V. O., Salakhutdinova M. Y., Epstein O. I. (2010). A comparison of the dynamics of S100B, S100A1, and S100A6 mRNA expression in Hippocampal CA1 area of rats during long-term potentiation and after low-frequency stimulation. *Cardiovascular Psychiatry and Neurology*.

[15] Vassilev L. T., Vu B. T., Graves B. (2004). In vivo activation of the p53 pathway by small-molecule antagonists of MDM2. *Science*.

[16] Huang B., Vassilev L. T. (2009). Reduced transcriptional activity in the p53 pathway of senescent cells revealed by the MDM2 antagonist nutlin-3. *Aging*.

[17] Carvajal L. A., Hamard P.-J., Tonnessen C., Manfredi J. J. (2012). E2F7, a novel target, is up-regulated by p53 and mediates DNA damage-dependent transcriptional repression. *Genes & Development*.

[18] Michael D., Oren M. (2003). The p53-Mdm2 module and the ubiquitin system. *Seminars in Cancer Biology*.

[19] Ofir-Rosenfeld Y., Boggs K., Michael D., Kastan M. B., Oren M. (2008). Mdm2 regulates p53 mRNA translation through inhibitory interactions with ribosomal protein L26. *Molecular Cell*.

[20] Nag S., Qin J., Srivenugopal K. S., Wang M., Zhang R. (2013). The MDM2-p53 pathway revisited. *Journal of Biomedical Research*.

[21] Moroni M. C., Hickman E. S., Denchi E. L. (2001). Apaf-1 is a transcriptional target for E2F and p53. *Nature Cell Biology*.

[22] Robles A. I., Bemmels N. A., Foraker A. B., Harris C. C. (2001). APAF-1 is a transcriptional target of p53 in DNA damage-induced apoptosis. *Cancer Research*.

[23] Miyashita T., Krajewski S., Krajewska M. (1994). Tumor suppressor p53 is a regulator of bcl-2 and bax gene expression in vitro and in vivo. *Oncogene*.

[24] Nakano K., Vousden K. H. (2001). PUMA, a novel proapoptotic gene, is induced by p53. *Molecular Cell*.

[25] Sax J. K., Fei P., Murphy M. E., Bernhard E., Korsmeyer S. J., El-Deiry W. S. (2002). BID regulation by p53 contributes to chemosensitivity. *Nature Cell Biology*.

[26] Hoffman W. H., Biade S., Zilfou J. T., Chen J., Murphy M. (2002). Transcriptional repression of the anti-apoptotic survivin gene by wild type p53. *The Journal of Biological Chemistry*.

[27] Löhr K., Möritz C., Contente A., Dobbelstein M. (2003). p21/CDKN1A mediates negative regulation of transcription by p53. *The Journal of Biological Chemistry*.

[28] Rouault J.-P., Falette N., Guehenneux F. (1996). Identification of BTG2, an antiproliferative p53-dependent component of the DNA damage cellular response pathway. *Nature Genetics*.

[29] Innocente S. A., Abrahamson J. L. A., Cogswell J. P., Lee J. M. (1999). p53 regulates a G_2_ checkpoint through cyclin B1. *Proceedings of the National Academy of Sciences of the United States of America*.

[30] Okamoto K., Beach D. (1994). Cyclin G is a transcriptional target of the p53 tumor suppressor protein. *The EMBO Journal*.

[31] Rother K., Kirschner R., Sänger K., Böhlig L., Mössner J., Engeland K. (2007). p53 downregulates expression of the G1/S cell cycle phosphatase Cdc25A. *Oncogene*.

[32] Clair S. S., Giono L., Varmeh-Ziaie S. (2004). DNA damage-induced downregulation of Cdc25C is mediated by p53 via two independent mechanisms: one involves direct binding to the cdc25C promoter. *Molecular Cell*.

[33] Badie C., Itzhaki J. E., Sullivan M. J., Carpenter A. J., Porter A. C. G. (2000). Repression of CDK1 and other genes with CDE and CHR promoter elements during DNA damage-induced G2/M arrest in human cells. *Molecular and Cellular Biology*.

[34] El-Deiry W. S., Tokino T., Waldman T. (1995). Topological control of p21WAF1/CIP1 expression in normal and neoplastic tissues. *Cancer Research*.

[35] Stott F. J., Bates S., James M. C. (1998). The alternative product from the human CDKN2A locus, p14(ARF), participates in a regulatory feedback loop with p53 and MDM2. *The EMBO Journal*.

[36] Matsui T., Katsuno Y., Inoue T. (2004). Negative regulation of Chk2 expression by p53 is dependent on the CCAAT-binding transcription factor NF-Y. *The Journal of Biological Chemistry*.

[37] Martoriati A., Doumont G., Alcalay M., Bellefroid E., Pelicci P. G., Marine J.-C. (2005). *Dapk1*, encoding an activator of a p19^ARF^-p53-mediated apoptotic checkpoint, is a transcription target of p53. *Oncogene*.

[38] Peterson E. J., Bögler O., Taylor S. M. (2003). p53-mediated repression of DNA methyltransferase 1 expression by specific DNA binding. *Cancer Research*.

[39] Ludes-Meyers J. H., Subler M. A., Shivakumar C. V. (1996). Transcriptional activation of the human epidermal growth factor receptor promoter by human p53. *Molecular and Cellular Biology*.

[40] Yu J., Baron V., Mercola D., Mustelin T., Adamson E. D. (2007). A network of p73, p53 and Egr1 is required for efficient apoptosis in tumor cells. *Cell Death and Differentiation*.

[41] Owen-Schaub L. B., Zhang W., Cusack J. C. (1995). Wild-type human p53 and a temperature-sensitive mutant induce Fas/APO-1 expression. *Molecular and Cellular Biology*.

[42] Müller M., Wilder S., Bannasch D. (1998). p53 activates the CD95 (APO-1/Fas) gene in response to DNA damage by anticancer drugs. *The Journal of Experimental Medicine*.

[43] Fukazawa T., Fujiwara T., Morimoto Y. (1999). Differential involvement of the CD95 (Fas/APO-1) receptor/ligand system on apoptosis induced by the wild-type p53 gene transfer in human cancer cells. *Oncogene*.

[44] Smith M. L., Chen I. T., Zhan Q. (1994). Interaction of the p53-regulated protein gadd45 with proliferating cell nuclear antigen. *Science*.

[45] Santhanam U., Ray A., Sehgal P. B. (1991). Repression of the interleukin 6 gene promoter by p53 and the retinoblastoma susceptibility gene product. *Proceedings of the National Academy of Sciences of the United States of America*.

[46] Zauberman A., Flusberg D., Haupt Y., Barak Y., Oren M. (1995). A functional p53-responsive intronic promoter is contained within the human *mdm2* gene. *Nucleic Acids Research*.

[47] Chen J., Sadowski I. (2005). Identification of the mismatch repair genes PMS2 and MLH1 as p53 target genes by using serial analysis of binding elements. *Proceedings of the National Academy of Sciences of the United States of America*.

[48] Scherer S. J., Maier S. M., Seifert M. (2000). p53 and c-Jun functionally synergize in the regulation of the DNA repair gene hMSH2 in response to UV. *The Journal of Biological Chemistry*.

[49] Moberg K. H., Tyndall W. A., Hall D. J. (1992). Wild-type murine p53 represses transcription from the murine c-myc promotor in a human glial cell line. *Journal of Cellular Biochemistry*.

[50] Morris G. F., Bischoff J. R., Mathews M. B. (1996). Transcriptional activation of the human proliferating-cell nuclear antigen promoter by p53. *Proceedings of the National Academy of Sciences of the United States of America*.

[51] Oda E., Ohki R., Murasawa H. (2000). Noxa, a BH3-only member of the Bcl-2 family and candidate mediator of p53-induced apoptosis. *Science*.

[52] Fiscella M., Zhang H., Fan S. (1997). Wip1, a novel human protein phosphatase that is induced in response to ionizing radiation in a p53-dependent manner. *Proceedings of the National Academy of Sciences of the United States of America*.

[53] Li C., Lin M., Liu J. (2004). Identification of PRC1 as the p53 target gene uncovers a novel function of p53 in the regulation of cytokinesis. *Oncogene*.

[54] Zhan M., Yu D., Liu J., Hannay J., Pollock R. E. (2005). Transcriptional repression of protein kinase C*α* via Sp1 by wild type p53 is involved in inhibition of multidrug resistance 1 P-glycoprotein phosphorylation. *The Journal of Biological Chemistry*.

[55] Stambolic V., MacPherson D., Sas D. (2001). Regulation of PTEN transcription by p53. *Molecular Cell*.

[56] Kho P. S., Wang Z., Zhuang L. (2004). p53-regulated transcriptional program associated with genotoxic stress-induced apoptosis. *Journal of Biological Chemistry*.

[57] Shiio Y., Yamamoto T., Yamaguchi N. (1992). Negative regulation of Rb expression by the p53 gene product. *Proceedings of the National Academy of Sciences of the United States of America*.

[58] Budanov A. V., Karin M. (2008). p53 target genes sestrin1 and sestrin2 connect genotoxic stress and mTOR signaling. *Cell*.

[59] Ohki R., Nemoto J., Murasawa H. (2000). Reprimo, a new candidate mediator of the p53-mediated cell cycle arrest at the G_2_ phase. *The Journal of Biological Chemistry*.

[60] Lin J., Yang Q., Yan Z. (2004). Inhibiting S100B restores p53 levels in primary malignant melanoma cancer cells. *The Journal of Biological Chemistry*.

[61] Brown L., Ongusaha P. P., Kim H.-G. (2007). CDIP, a novel pro-apoptotic gene, regulates TNF*α*-mediated apoptosis in a p53-dependent manner. *The EMBO Journal*.

[62] Zhao R., Gish K., Murphy M. (2000). Analysis of p53-regulated gene expression patterns using oligonucleotide arrays. *Genes & Development*.

[63] Lopez C. D., Ao Y., Rohde L. H. (2000). Proapoptotic p53-interacting protein 53BP2 is induced by UV irradiation but suppressed by p53. *Molecular and Cellular Biology*.

[64] Harmes D. C., Bresnick E., Lubin E. A. (2003). Positive and negative regulation of deltaN-p63 promoter activity by p53 and deltaN-p63-alpha contributes to differential regulation of p53 target genes. *Oncogene*.

[65] Grob T. J., Novak U., Maisse C. (2001). Human ΔNp73 regulates a dominant negative feedback loop for TAp73 and p53. *Cell Death and Differentiation*.

[66] Chen X., Zheng Y., Zhu J., Jiang J., Wang J. (2001). p73 is transcriptionally regulated by DNA damage, p53, and p73. *Oncogene*.

[67] Israeli D., Tessler E., Haupt Y. (1997). A novel p53-inducible gene, PAG608, encodes a nuclear zinc finger protein whose overexpression promotes apoptosis. *The EMBO Journal*.

[68] Lisachev P. D., Pustylnyak V. O., Shtark M. B., Epstein O. I. (2013). Induction of S100B gene expression in long-term potentiation in the hippocampal CA1 field depends on activity of NMDA receptors. *Bulletin of Experimental Biology and Medicine*.

[69] Sokolova O. O., Shtark M. B., Lisachev P. D., Pustylnyak V. O., Pan I. R., Epstein O. I. (2009). Expression of S100B and S100A6 genes during long-term posttetanic potentiation in the hippocampus. *Bulletin of Experimental Biology and Medicine*.

[70] Huang Y. Y., Kandel E. R. (1994). Recruitment of long-lasting and protein kinase A-dependent long-term potentiation in the CA1 region of hippocampus requires repeated tetanization. *Learning & Memory*.

[71] Biderman L., Manley J. L., Prives C. (2012). Mdm2 and MdmX as regulators of gene expression. *Genes & Cancer*.

[72] Park C. S., Gong R., Stuart J., Tang S.-J. (2006). Molecular network and chromosomal clustering of genes involved in synaptic plasticity in the hippocampus. *Journal of Biological Chemistry*.

[73] Ploski J. E., Park K. W., Ping J., Monsey M. S., Schafe G. E. (2010). Identification of plasticity-associated genes regulated by Pavlovian fear conditioning in the lateral amygdala. *Journal of Neurochemistry*.

[74] Ryan M. M., Mason-Parker S. E., Tate W. P., Abraham W. C., Williams J. M. (2011). Rapidly induced gene networks following induction of long-term potentiation at perforant path synapses in vivo. *Hippocampus*.

[75] Ryan M. M., Ryan B., Kyrke-Smith M. (2012). Temporal profiling of gene networks associated with the late phase of long-term potentiation in vivo. *PLoS ONE*.

[76] Lein E. S., Zhao X., Gage F. H. (2004). Defining a molecular atlas of the hippocampus using DNA microarrays and high-throughput *in situ* hybridization. *The Journal of Neuroscience*.

[77] Leonardo E. D., Richardson-Jones J. W., Sibille E., Kottman A., Hen R. (2006). Molecular heterogeneity along the dorsal-ventral axis of the murine hippocampal CA1 field: a microarray analysis of gene expression. *Neuroscience*.

[78] Maggio N., Segal M. (2007). Unique regulation of long term potentiation in the rat ventral hippocampus. *Hippocampus*.

[79] Riley T., Sontag E., Chen P., Levine A. (2008). Transcriptional control of human p53-regulated genes. *Nature Reviews Molecular Cell Biology*.

[80] Miyamoto E. (2006). Molecular mechanism of neuronal plasticity: induction and maintenance of long-term potentiation in the hippocampus. *Journal of Pharmacological Sciences*.

[81] Sukhatme V. P., Cao X. M., Chang L. C. (1988). A zinc finger-encoding gene coregulated with *c-fos* during growth and differentiation, and after cellular depolarization. *Cell*.

[82] Quiñones A., Dobberstein K. U., Rainov N. G. (2003). The egr-1 gene is induced by DNA-damaging agents and non-genotoxic drugs in both normal and neoplastic human cells. *Life Sciences*.

[83] de Belle I., Huang R.-P., Fan Y., Liu C., Mercola D., Adamson E. D. (1999). P53 and Egr-1 additively suppress transformed growth in HT1080 cells but Egr-1 counteracts p53-dependent apoptosis. *Oncogene*.

[84] Pignatelli M., Luna-Medina R., Pérez-Rendón A., Santos A., Perez-Castillo A. (2003). The transcription factor early growth response factor-1 (EGR-1) promotes apoptosis of neuroblastoma cells. *The Biochemical Journal*.

[85] Bálint E., Bates S., Vousden K. H. (1999). Mdm2 binds p73*α* without targeting degradation. *Oncogene*.

